# DNA Vaccine Development at Pre- and Post-Operation Warp Speed

**DOI:** 10.3390/vaccines8040737

**Published:** 2020-12-04

**Authors:** Karl Ljungberg, Maria Isaguliants

**Affiliations:** 1 Eurocine Vaccines AB, Fogdevreten 2, 17165 Solna, Sweden; karl.ljungberg@eurocine-vaccines.com; 2 Department of Microbiology, Tumor and Cell Biology, Karolinska Institutet, 171 77 Stockholm, Sweden; 3 Department of Research, Riga Stradins University, LV-1007 Riga, Latvia; 4 N.F. Gamaleya Research Center for Epidemiology and Microbiology, 123098 Moscow, Russia; 5 M.P. Chumakov Federal Scientific Center for Research and Development of Immune-and-Biological Products of Russian Academy of Sciences, 108819 Moscow, Russia

DNA is a rapidly developing vaccine platform for combatting cancer, infectious and noninfectious diseases. Plasmid DNA used as immunogens encode proteins to be synthesized in the cells of the vaccine recipients. Introduction of DNA vaccines into the host induces antibody and cellular responses against the encoded protein. In this way, the induction of immune response mimics the events occurring during natural infection with an intracellular pathogen. There are a few distinct ways in which the vaccine antigen can be processed and presented, which shape the resulting immune response and which can be manipulated. Characteristically, the antigen synthesized within the host cell is processed by the proteasome, loaded onto, and presented on MHC Class I molecules. Processing can be re-routed to the lysosome, or the immunogen can be secreted for further presentation on MHC Class II. Vaccine efficacy is also highly dependent on DNA delivery. DNA immunogens are often administered by intramuscular or intradermal injections, but the immune response can be significantly enhanced by subsequent electroporation of the injection site, which enhances the delivery up to 1000-fold, thereby facilitating dose sparing. Other techniques may also be employed. For instance, noninvasive introduction by biolistic devices such as gene guns and biojectors, skin applications with plasters and microneedles/chips, sonication, magnetofection, and even tattooing has been shown to improve the efficacy of delivery. The debate regarding the pros and cons of different routes of delivery is intense but the answer to which route of administration is better for a DNA vaccine is too complex to give a straightforward answer. It depends on multiple factors such as the choice of antigen and vector, expressing tissues and cells, and the disease it targets. A number of studies have compared the effect of delivery methods on the level of immunogen expression, and the magnitude and specificity of the resulting immune response. According to some, the delivery route determines the immunogenic performance; according to others, it can modulate the level of response but not its specificity or polarity. All in all, research on the optimization of DNA vaccine design, delivery, and immunogenic performance has led to a marked increase in their efficacy in larger species and humans.

This Special Issue describes the continuing efforts to increase the potency of DNA vaccines by manipulating plasmid DNA, adding adjuvants or immunomodulators (also delivered in the form of plasmid DNA), and by using a wide panel of novel delivery systems. These efforts are described in seven experimental papers and four reviews.

When we launched the “Advances in DNA Vaccines” Special Issue, little did we know that the world would face a new infectious disease threat just months after the submission deadline, a threat which would change the entire game plan for vaccine development. The current SARS-CoV-2 pandemic has forced vaccinology into a new intense phase of development, transformation, and innovation. Hit by the COVID-19 epidemics, the world has come to realize the importance of efficient vaccines against viral infections. It is comforting that the contributing authors were working hard in this field before the epidemic broke out: all publications of this issue focus on the development of vaccines against viral infections, such as HIV by Kilpelainen et al. [1], Louis et al. [2], and Akulova et al. [3]; human hepatitis C virus by Masalova et al. [4]; Ebola virus by Bazhan et al. [5]; Zika virus [2]; influenza by Hinkula et al. [6] and Louis et al. [2]; and Epstein–Barr virus by Wojtak et al. [7].

The majority of studies presented in this issue used different forms of plasmid DNA: genes optimized for expression and/or consensus immunogens [2,3,7], polyepitope constructs [5], or plasmids with viral enhancers increasing protein expression and thus allowing one to reduce the DNA dose (Chapman & Rybicki [8]). A still more efficient way to ensure high-level expression of the immunogen is to use RNA/DNA layered alphavirus vectors, which provide a superior expression of immunogens in comparison with conventional plasmid DNA technology (Lundstrom [9]). Besides, immunization with alphavirus DNA vectors elicits an immune response compared with the conventional plasmid at a 1000-fold lower DNA dose, allowing for considerable vaccine sparing [9].

In most of the studies presented in this Special Issue, plasmid DNA was introduced by intramuscular or intradermal injections, with or without subsequent electroporation. Two more sophisticated ways of indirect delivery of DNA encoding immunogens presented include immunization with recombinant BCG as a vehicle to express HIV-1 and SIV antigens [1] and by mesenchymal stem cells made to express nonstructural proteins of hepatitis C virus [4]. In both cases, delivery using bacterial or eukaryotic cells as a vehicle allowed the researchers to significantly increase the cellular response against viral antigens as compared with immunization with naked plasmid DNA.

A recent development is the use of plasmid DNA to encode an adjuvant; for instance, a cytokine or another noncytokine immunomodulator with the power to enhance and shape the immune response. In this Special Issue, we see demonstrations of the efficient use of an immunomodulatory plasmid DNA encoding IL-36 gamma, giving a boost to an immune response against HIV, Zika virus, and influenza in mice [2]. The review by Shrestha & Grubor-Bauk gives an excellent example of the use of a novel cytolytic platform of DNA immunization based on truncated mouse perforin [10], whereas Hinkula et al. [6] use a plasmid DNA encoding the TLR5 ligand flagellin as an immunomodulator which, if administered together with formalin-inactivated whole influenza A vaccine, increases the antibody response 200-fold, hemagglutination inhibition (HAI) titer by 100-fold, and the cellular response against flu by 40-fold.

An overview of the experimental papers in this Special Issue reveals that DNA vaccines of the pre-COVID-19 era were not yet mature enough to be an instrument of human vaccination, as six out of seven experimental papers describe the immunization of mice; only one, by Akulova et al., describes the results of the Phase II clinical trial of a HIV DNA vaccine candidate [3]. However, today, the situation is rapidly changing.

The spread of the SARS-CoV-2 virus and the COVID-19 disease has triggered an unprecedented surge in vaccine research and development, as well as attracting an unsurpassed amount of funding in a very limited time—and for good reasons. Worldwide infections with SARS-CoV-2 are increasing rapidly and, at the time of writing this editorial, approach 40 million diagnosed cases; the actual number of infections is much higher. Over one million deaths have resulted from a disease that did not exist for us a year ago. This new virus disseminates in a way that has been impossible to predict; traditional countermeasures such as hygiene, physical distancing, various levels of public lockdowns, and quarantines have proven to be insufficient, highlighting the urgent need for prophylactic vaccine(s) to be applied worldwide as the only way to stop viral spread.

The ongoing COVID-19 crisis has led to a very rapid development of vaccine candidates from academia and small and medium-sized biotech companies, as well as pharmaceutical giants. Now, nine months down the road, several candidates have already gone through discovery, preclinical testing demonstrating immunogenicity, and, in some cases, efficacy, process development, toxicity testing, production and recruitment for and completion of Phase 1 safety and immunogenicity studies. The most advanced candidates have already proceeded into Phase 2 and 3 testing and scaling up; preparation for large-scale production is well under way. It is hard to comprehend what an achievement this really is and it is important to recognize that a lot of the progress has been made through performing multiple steps in the vaccine (Research & Development) R&D process in parallel and at great financial risk: in the case where a vaccine candidate does not show the expected immunogenicity or safety, for instance, the resources invested in that candidate will be lost. In a traditional stepwise approach to vaccine development, unsuccessful candidates would have been deselected at an earlier stage and the financial risks would have been mitigated.

It is important to emphasize that this approach is only made possible through numerous initiatives both from states, not-for-profit organizations such as the Coalition for Epidemic Preparedness Innovations (CEPI) and the Bill and Melinda Gates Foundation, and private equity alike. One such initiative is Operation Warp Speed, to which the US Congress has directed almost 10 billion USD. Operation Warp Speed aims to deliver 300 million doses of a safe, effective vaccine for COVID-19 by January 2021 as part of a broader strategy to accelerate the development, manufacturing, and distribution of COVID-19 vaccines, therapeutics, and diagnostics (https://www.hhs.gov/about/news/2020/06/16/fact-sheet-explaining-operation-warp-speed.html).

At no time in history has biomedical research moved forward at such a high pace. The research community, the pharmaceutical sector, and funding agencies have responded to the threat with dedication. Moreover, the dissemination of data has never been faster. Rapid publication of data has been a key factor in the battle against the virus and the role of preprint servers such as biorxiv and biomedrxiv cannot be underestimated. For instance, the genome sequence of SARS-CoV-2 was published online less than two weeks after the Wuhan Municipal Health Commission in Wuhan City, China, reported a cluster of 27 pneumonia cases of unknown etiology, and only one day after the Chinese CDC reported that a novel coronavirus had been detected as the causative agent of COVID-19 (https://virological.org/t/novel-2019-coronavirus-genome/319/26). Since then, COVID-19 research has exploded; never before have studies on a previously undescribed disease and disease-causing agent provided so much knowledge in such a short time. The remarkable progress in synthetic biology and manufacturing of DNA sequences, based on the availability of sequences, not biological materials, enabled the rapid development of vaccine candidates against COVID-19. We see this as an argument in favor of the synthetic nucleic acid-based vaccines.

Thus, it is not surprising that some of the most advanced vaccine candidates against COVID-19 represent novel previously unlicensed technology platforms exploiting synthetic genes, such as adenovirus vector platforms (e.g., Astra Zeneca, CanSino Biologics, Sputnik-V; https://clinicaltrials.gov/ct2/show/NCT04437875), mRNA (e.g., Moderna, Sanofi, Curevac, Pfizer/BioNTech), and, of course, DNA, such as INO-4800 by Inovio, thought to be the furthest ahead among four DNA-based vaccines that have started human testing for COVID-19 (https://clinicaltrials.gov/ct2/show/NCT04336410). A concise review by Liu in this Special Issue underpins pre-COVID-19 era efforts to make DNA vaccines more efficient, comparing them with similar efforts made for mRNA vaccines [11]. At the moment, the reader may get an impression that the mRNA vaccines have the upper hand, specifically referring to the many advanced mRNA COVID-19 vaccine candidates in clinical trials. However, the INO-4800 DNA vaccine also showed promise in a Phase 1 trial: INO-4800 was safe and well-tolerated and induced immune responses in a majority of participants (https://www.fdanews.com/articles/199278-inovios-covid-19-vaccine-trial-placed-on-partial-hold). Taking this stand, we are far from expressing DNA vaccine pessimism. It is likely that the emergence of RNA vaccines as a viable vaccine modality and the technological developments that are driven by this field may also impact the development of new DNA vaccine and related technologies. Given the tremendous need for: (i) high speed in vaccine development relying on the availability of sequence information, (ii) the ease and speed of vaccine production using already established platforms, and (iii) no cold chain to allow for worldwide distribution, which are provided by DNA vaccines, one can be sure that the first vaccines of this type will soon see licensure for human use and find clinical application abroad.

When the smoke of the battle against COVID-19 and the race for an effective vaccine settles, we will face a new normal where the dissemination of SARS-CoV-2 is hopefully controlled, although the way we interact with each other and the way we work would be changed once and forever. People would gradually forget the times of hardship that we are currently facing. It may happen that our collective memory will be shorter than the T cell memory. Nevertheless, we hope that the insights gained and lessons learned in vaccinology will prevail and that investments will continue to be made in this domain to further advance vaccinology and prevention of infectious diseases in all corners of the world, since every dollar invested in vaccinology has an enormous potential return in saving lives and improving peoples’ health. As scientists and vaccine advocates, we have to act to preserve this momentum. One day, we will be out of the COVID-19 pandemic, but we can be sure that new challenges will appear on the horizon. We have to keep up the pace to be prepared.
